# Growth substrate limitation enhances anaerobic arsenic methylation by *Paraclostridium bifermentans* strain EML

**DOI:** 10.1128/aem.00961-24

**Published:** 2024-11-08

**Authors:** Jiangtao Qiao, Hugo Sallet, Karin Lederballe Meibom, Rizlan Bernier-Latmani

**Affiliations:** 1 Environmental Microbiology Laboratory, School of Architecture, Civil and Environmental Engineering, École Polytechnique Fédérale de Lausanne (EPFL), Lausanne, Switzerland; 2 Guangdong Key Laboratory of Integrated Agro-environmental Pollution Control and Management, Institute of Eco-environmental and Soil Sciences, Guangdong Academy of Sciences, Guangzhou, China; 3 National-Regional Joint Engineering Research Center for Soil Pollution Control and Remediation in South China, Guangzhou, China; Washington University in St. Louis, St. Louis, Missouri, USA

**Keywords:** anaerobic arsenic methylation, MMAs(III), *arsM *gene transcript, anaerobic co-culture, *arsP*, *E. coli *MG1655

## Abstract

Microbial arsenic methylation is established as a detoxification process under aerobic conditions (converting arsenite to monomethylated arsenate) but is proposed to be a microbial warfare strategy under anoxic conditions due to the toxicity of its main product, monomethylarsonous acid (MMAs(III)). Here we leveraged a paddy soil-derived anaerobic arsenic methylator, *Paraclostridium bifermentans* strain EML, to gain insights into this process. Strain EML was inoculated into a series of media involving systematic dilutions of Reinforced Clostridial Broth (RCB) with 25 µM arsenite to assess the impact of growth substrate concentration on arsenic methylation. Growth curves evidenced the sensitivity of strain EML to arsenite, and arsenic speciation analysis revealed the production of MMAs(III). Concentrations of MMAs(III) and arsenic methylation gene (*arsM*) transcription were found to be positively correlated with RCB dilution, suggesting that substrate limitation enhances *arsM* gene expression and associated anaerobic arsenic methylation. We propose that growth substrate competition among microorganisms may also contribute to an increase in anaerobic arsenic methylation. This hypothesis was further evaluated in an anaerobic co-culture system involving strain EML and either wild-type *Escherichia coli* K-12 MG1655 (WT) or *E. coli* expressing the MMAs(III)-resistance gene (*arsP*) (ArsP *E. coli*). We observed increased MMAs(III) production in the presence of *E. coli* than its absence and growth inhibition of WT *E. coli* to a greater extent than ArsP *E. coli*, presumably due to the MMAs(III) produced by strain EML. Collectively, our findings suggest an ecological role for anaerobic arsenic methylation, highlighting the significance of microbe-microbe competition and interaction in this process.

**IMPORTANCE:**

Microbial arsenic methylation is highly active in rice paddy fields under flooded conditions, leading to increased accumulation of methylated arsenic in rice grains. In contrast to the known detoxification process for aerobic arsenic methylation, the ecological role of anaerobic arsenic methylation remains elusive and is proposed to be an antibiotic-producing process involved in microbial warfare. In this study, we interrogated a rice paddy soil-derived anaerobic arsenic-methylating bacterium, *Paraclostridium bifermentans* strain EML, to explore the effect of growth substrate limitation on arsenic methylation in the context of the microbial warfare hypothesis. We provide direct evidence for the role of growth substrate competition in anaerobic arsenic methylation *via* anaerobic prey-predator co-culture experiments. Moreover, we demonstrate a feedback loop, in which a bacterium resistant to MMAs(III) enhances its production, presumably through enhanced expression of *arsM* resulting from substrate limitation. Our work uncovers the complex interactions between an anaerobic arsenic methylator and its potential competitors.

## INTRODUCTION

Microbial transformations play an important role in the biogeochemical cycling of arsenic (As) in the environment, and include reduction, oxidation, thiolation, methylation, and demethylation of inorganic and organic As (1–4). These reactions impact the mobility, bioavailability, and toxicity of As compounds (1–3, 5, 6). In recent years, microbial transformations of As in paddy soil have drawn increasing attention because of the potential health risk of dietary exposure to As from rice-containing products (7, 8). For instance, organic As (in particular dimethyl arsenate, DMAs(V)) is commonly detected in rice grains, along with inorganic As (9, 10). As DMAs(V) is much less toxic than arsenite, accumulation of DMAs(V) in rice grains largely reduces its toxicity to humans. However, there is evidence of a correlation between DMAs(V) accumulation in rice grains and rice straight-head disease, a condition that decreases rice crop yields (11, 12). In addition, because analyzing As in rice requires digestion, there is a concern that the highly toxic dimethylated monothioarsenate (DMMTA) may be transformed to and measured as DMAs(V) (13), unknowingly overlooking a potential threat to human health.

Arsenic methylation is a microbially mediated process involving the transformation of inorganic trivalent As (iAs(III)) into mono-, di-, and trimethylated As compounds and is catalyzed by *S*-adenosyl-methionine methyltransferase (ArsM in prokaryotes) (14–16). Generally, As methylation occurring under oxic conditions is proposed as an iAs(III)-detoxifying process because although more toxic As compounds (monomethylarsonous acid (MMAs(III) and dimethylarsinous acid (DMAs(III)) are produced, they are rapidly oxidized in the presence of O_2_ to their less toxic pentavalent counterparts (monomethylarsonic acid (MMAs(V) and dimethylarsinic acid (DMAs(V)) (16). This paradigm is supported by the fact that the heterologous expression of the *arsM* gene conferred As(III) resistance to an As(III)-sensitive *Escherichia coli* strain under aerobic conditions (16, 17).

By contrast, detoxification is unlikely to be the ecological function of As-methylators inhabiting anoxic environments since organic As products are present in their trivalent forms. Interestingly, the evolutionary history of the *arsM* gene predicts its emergence during the anoxic Archaean era, when MMAs(III) would have been chemically stable (18, 19). This finding suggests that the original function of MMAs(III) could have been to serve as a primitive antibiotic (20, 21). A closer examination of As methylation capability among previously reported and available pure anaerobic strains uncovered either the negligible methylation efficiency or the fortuitous methylation of As upon cell lysis and the release of methyltransferases (22), making it challenging to decipher the physiological function of anaerobic As methylation. At present, confirmed and available anaerobic As-methylating bacteria come from the genus *Paraclostridum* (23, 24). Here, we will study *Paraclostridium bifermentans* strain EML (henceforth strain EML), a fermenter isolated from paddy soil in Vietnam (24).

In microbial communities, competitive phenotypes (including the production of antibiotics) can arise as a consequence of limited resources (e.g., nutrients and space) (25). Soils represent an ecosystem in which many microorganisms compete for scarce resources, thus competition is widespread (26). We hypothesize that competition for resources may boost As methylation by strain EML, conferring it an advantage over its competitors. In this study, we aim to investigate the effect of growth substrate limitation on As methylation by strain EML in the context of the microbial warfare hypothesis for anaerobic As methylation. Furthermore, we probe direct microbial interaction/inhibition between strain EML and potential competitors (*Escherichia coli* MG1655 either the wild-type strain (WT) or one engineered to express the MMAs(III)-resistance gene (*arsP*)). From an ecological point of view, it is reasonable to expect enhanced As methylation under substrate-limiting conditions as a response to resource competition. According to our hypothesis, strain EML would increase the production of toxic MMAs(III) under growth substrate-limited conditions to thwart other microorganisms competing for the same resources. Although DMAs(III) might also function as an antibiotic under anaerobic conditions, we focus on MMAs(III) in this study due to the analytical limitations associated with DMAs(III).

## RESULTS

### iAs(III) inhibits strain EML growth

**Fig 1:**
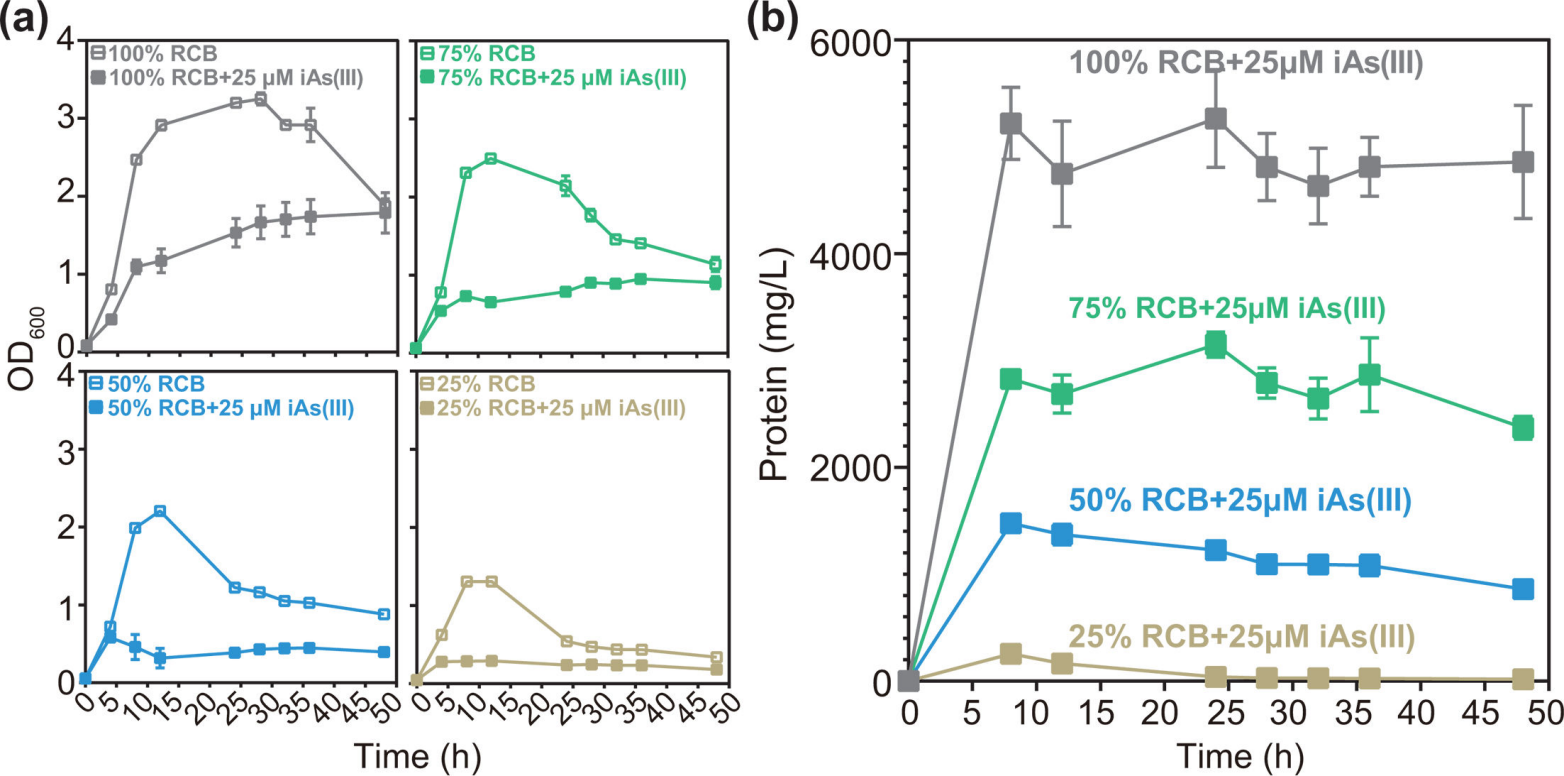
Growth curves [optical density (OD_600_] (**a**) and total protein content (**b**) of *Paraclostridium bifermentans* strain EML in anaerobic dilutions of RCB (100%, 75%, 50%, or 25% RCB) in the presence or absence of 25 µM iAs(III). Data are shown as mean values with error bars. Individual values for each biological triplicate are included in Data Table 1.

Strain EML was grown under anoxic conditions with various concentrations of Reinforced Clostridial Broth (RCB), including 100%, 75%, 50%, or 25% RCB, in the presence and absence of 25 µM iAs(III). Growth curves reveal that, in the absence of iAs(III), strain EML exhibited rapid growth, reaching the mid-exponential growth phase within approximately 8 h (Fig. 1). By contrast, in the presence of iAs(III), strain EML displayed reduced growth rates and an earlier onset of stationary phase, particularly under low growth-substrate conditions (50% and 25% RCB) (Fig. 1). More precisely, at early stationary phase under iAs(III)-treated conditions, growth declined by 48.7% ± 7.2%, 68.3% ± 1.8%, 77.1% ± 7.6%, and 78.0% ± 0.4% relative to the corresponding no-iAs(III) controls in 100%, 75%, 50%, and 25% RCB, respectively (Fig. 1). Although strain EML harbors a gene encoding an iAs(III) efflux permease (*acr3*) (Fig. S1), we hypothesize that it may not pump out intracellular iAs(III) sufficiently fast to preclude toxicity.

### Impact of substrate concentration on MMAs(III) production by strain EML

To investigate the dynamics of iAs(III) transformation during the anaerobic growth of strain EML, time-dependent changes in As speciation were measured both in the culture solution (aqueous) and within cells (soluble intracellular). The analysis of aqueous As species indicated that MMAs(III) was gradually generated by strain EML. The concentration of MMAs(III) increased markedly during the exponential growth phase (0–12 h), ranging from 0.14 ± 0.01 to 0.38 ± 0.06 µM across all tested culture conditions. This was followed by a moderate plateau between 24 and 48 h, correlating with the post-stationary to death phases, with MMAs(III) levels reaching 0.18 ± 0.01 to 0.72 ± 0.10 µM, in the order of 100% RCB >75% RCB >50% RCB >25% RCB (Fig. S2a through d). Notably, sterile RCB amended with 25 µM iAs(III) exhibited no transformation, as the iAs(III) concentration remained stable at approximately 25 µM from the start to the end of the incubation (Table S1).

**Fig 2:**
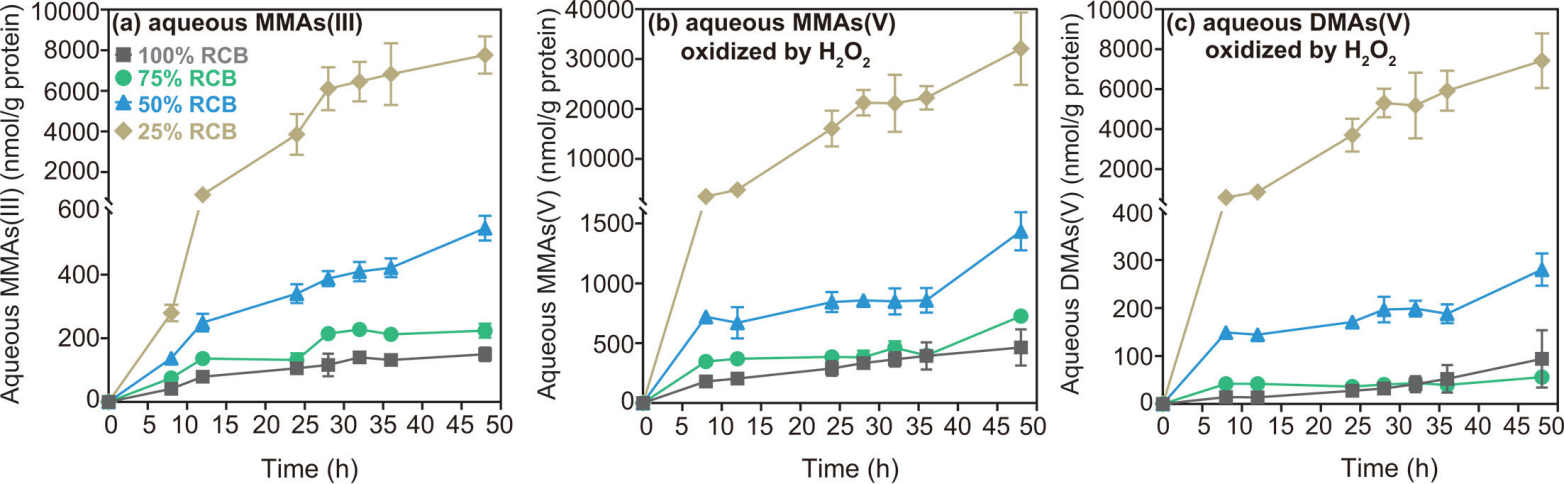
Time-dependent concentrations of protein-normalized aqueous As species in anaerobic RCB dilutions (100%, 75%, 50%, or 25% RCB) inoculated with *Paraclostridium bifermentans* strain EML and 25 µM iAs(III). (**a**) MMAs(III) (no oxidation), (**b**) MMAs(V) (post-oxidation), and (**c**) DMAs(V) (post-oxidation). Individual values for each biological replicate can be found in Data Tables 2 and 19.

As expected, strain EML exhibited variable growth rates across different RCB dilutions (Fig. 1), confounding the interpretation of whether growth substrate levels affected the extent of As methylation. Normalization of methylated As (both oxidized and non-oxidized forms) to protein concentration (Fig. 2) revealed a trend in MMAs(III) levels (Fig. 2a) and in MMAs(V) or DMAs(V) across RCB dilutions (Fig. 2b and c). Specifically, the normalized concentration of MMAs(III) decreased in the order of 25% RCB >50% RCB >75% RCB >100% RCB (Fig. 2). The highest protein-normalized MMAs(III) concentration was observed in the most diluted condition (25% RCB, 7,766 ± 919 nmol/g protein), representing increases of approximately 14-fold (546 ± 39 nmol/g protein), 34-fold (224 ± 22 nmol/g protein), and 51-fold (150 ± 21 nmol/g protein) compared to the 50%, 75%, and 100% RCB conditions, respectively (Fig. 2a). Similar patterns were also observed for the oxidized samples, with the highest protein-normalized MMAs(V) and DMAs(V) concentrations detected in the highest RCB dilution (25% RCB) (Fig. 2b and c). Additionally, soluble intracellular As analysis further confirms that strain EML accumulates substantial amounts of intracellular iAs(III) and MMAs(V) during anaerobic As methylation, particularly under the highest RCB dilution (25% RCB) (Fig. S3).

### Chemical transformation of MMAs(III) in biological media

While MMAs(III) is produced by strain EML, its chemical stability is often limited, even under anoxic conditions. This limitation is due to side chemical reactions such as thiolation, which can lead to an underestimation of the concentration of MMAs(III) produced. We tested this stability by amending anoxic RCB (100%–25%) or anoxic spent RCB (in which strain EML had grown) with 3 µM MMAs(III) and documented its significant disappearance in solution after 24 h. Surprisingly, MMAs(III) stability was highest in 100% RCB and lowest in 25% RCB, suggesting an inverse relationship between MMAs(III) stability and RCB dilution (Fig. S4 and S5). This limited chemical stability of MMAs(III) in both fresh and spent RCB medium suggests that the actual MMAs(III) production may be underestimated in all conditions, with more pronouced underestimation in the more dilute RCB media.

The observed disappearance of MMAs(III) from solution upon amendment to RCB medium was puzzling, leading us to hypothesize the formation of methylated-thiolated As species, such as monomethyldithioarsenate, MMDTAs(V), some of which might not be detectable using our analytical methods. To explore this hypothesis, we conducted As speciation analysis following the oxidation of trivalent As species using 10% (vol/vol) H_2_O_2_ (27–29). This oxidation step is expected to transform MMAs(III) into MMAs(V) quantitatively and to oxidize the thiol group in MMDTAs (and other monomethylated-thiolated species) to sulfate, releasing it and resulting in MMAs(V) as the final product. Indeed, post-oxidation analysis revealed a higher concentration of MMAs(V) compared to MMAs(III) prior to oxidation (Fig. 2; Fig. S2e through h, S6, and S7; Text SR1), suggesting the conversion of additional monomethylated As compounds, other than MMAs(III), to MMAs(V).

Direct evidence of the chemical transformation of MMAs(III) was demonstrated through the reaction of sulfide with MMAs(III), which resulted in the retention of a portion of the As species by the column (Fig. S8a). Furthermore, following oxidation with H_2_O_2_, the entire As inventory was recovered as MMAs(V) (Fig. S8b). Based on these observations, we propose that compounds, such as MMDTAs(V) or others, are formed *via* the chemical reaction of MMAs(III) with reduced sulfur compounds present in the growth medium. These reduced chemical species are likely retained by the HPLC column. When samples are oxidized prior to measurement, the monomethylated-thiolated species are oxidized to MMAs(V), which can then be readily eluted. Consequently, in oxidized samples, the MMAs(V) concentration corresponds to the sum of both MMAs(III) and the monomethylated-thiolated As species.

### *arsM* gene transcription under variable substrate conditions

**Fig 3:**
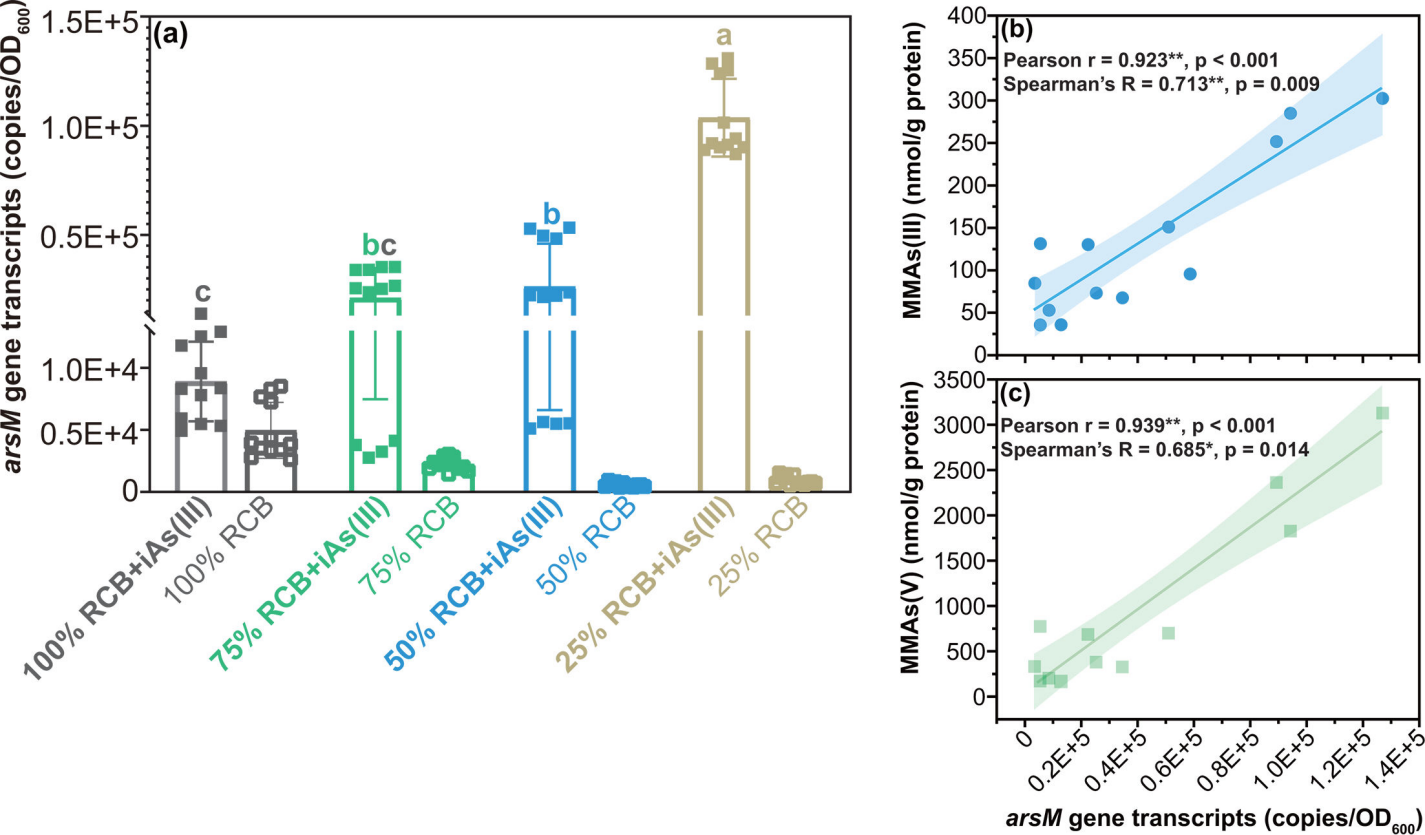
(**a**) Transcripts of *arsM* gene of *Paraclostridium bifermentans* strain EML in anaerobic RCB dilutions (100%, 75%, 50%, or 25% RCB) in the presence and absence of 25 µM iAs(III) at 8 h of incubation. (**b** and **c**) Correlation analysis of *arsM* gene transcripts and concentrations of MMAs(III) (no oxidation), and MMAs(V) (post-oxidation) at 8 h of incubation. Different letters showed significant differences between RCB dilutions at *P* < 0.05. Individual values for each biological replicate are shown in Data Tables 3 and 20.

To investigate whether the transcription of the gene responsible for As(III) methylation (*arsM*) is influenced by growth substrate concentration, we quantified gene expression using reverse transcription quantitative PCR (RT-qPCR). Initially, we attempted relative expression analysis and evaluated the expression stability of eight potential reference genes (Table S2) using qBase plus software. Unfortunately, we could not identify an optimal number of reference genes due to the relatively high variability among sequential normalization factors (geNorm V > 0.15), and the expression stability achieved was limited (0.5 < average geNorm M ≤ 1.0) (Fig. S9). We suspect that the considerable variation in growth rates resulting from RCB dilutions and the presence of iAs(III) markedly impacted gene expression, including that of the so-called housekeeping genes. Consequently, we opted for absolute quantification. We standardized the biomass (OD_600_) of strain EML, obtained through variable RCB dilutions, to approximately the same value prior to RNA extraction to eliminate potential biases related to biomass in RNA extraction and reverse transcription. As expected, the transcripts of strain EML *arsM* gene (adjusted to OD_600_) were significantly higher (*P* < 0.05) in the presence of iAs(III) compared to the controls without iAs(III) (Fig. 3a). Among the treatments with iAs(III), we observed that *arsM* gene transcript copy numbers exhibited an opposing relationship with substrate concentration: 25% RCB >50% RCB >75% RCB >100% RCB (Fig. 3a). The highest number of *arsM* transcripts was detected in the most dilute medium (25% RCB +iAs(III), 1.04E + 05 ± 2.05E + 04 copies/OD_600_), which was approximately 4, 5, and 12 times greater than in the treatments of 50% RCB +iAs(III) (2.63E + 04 copies/OD_600_), 75% RCB +iAs(III) (2.11E + 04 copies/OD_600_), and 100% RCB +iAs(III) (8.90E + 03 copies/OD_600_), respectively (Fig. 3a). This result is robust because, as in the absence of iAs(III), the trend is reversed, with *arsM* expression being highest under the no dilution (100% RCB) condition (Fig. 3a). We attribute this trend to imperfect normalization of *arsM* expression, as we presume that the expression of the *arsM* gene in all dilutions should remain consistent across all dilutions without iAs(III). This discrepancy may arise from differences in expression between cells growing in substrate-replete and substrate-depleted conditions. However, these biases actually reinforce the findings reported in the presence of iAs(III), as they would tend to underestimate *arsM* expression in higher dilution conditions, whereas our results indicate the highest transcript numbers in those conditions.

Furthermore, correlation analysis revealed a significant positive correlation (*P* < 0.05) between *arsM* gene transcripts and the concentrations of aqueous MMAs(III) (no oxidation) (Fig. 3b) and aqueous MMAs(V) (post-oxidation) (Fig. 3c). This relationship was further corroborated by the second experimental method employed for absolute transcript quantification, which utilized the same amount of total RNA for reverse transcripts, independent of the biomass amount used for extraction (Fig. S10).

### Impact of strain EML on *E. coli* growth rate

**Fig 4:**
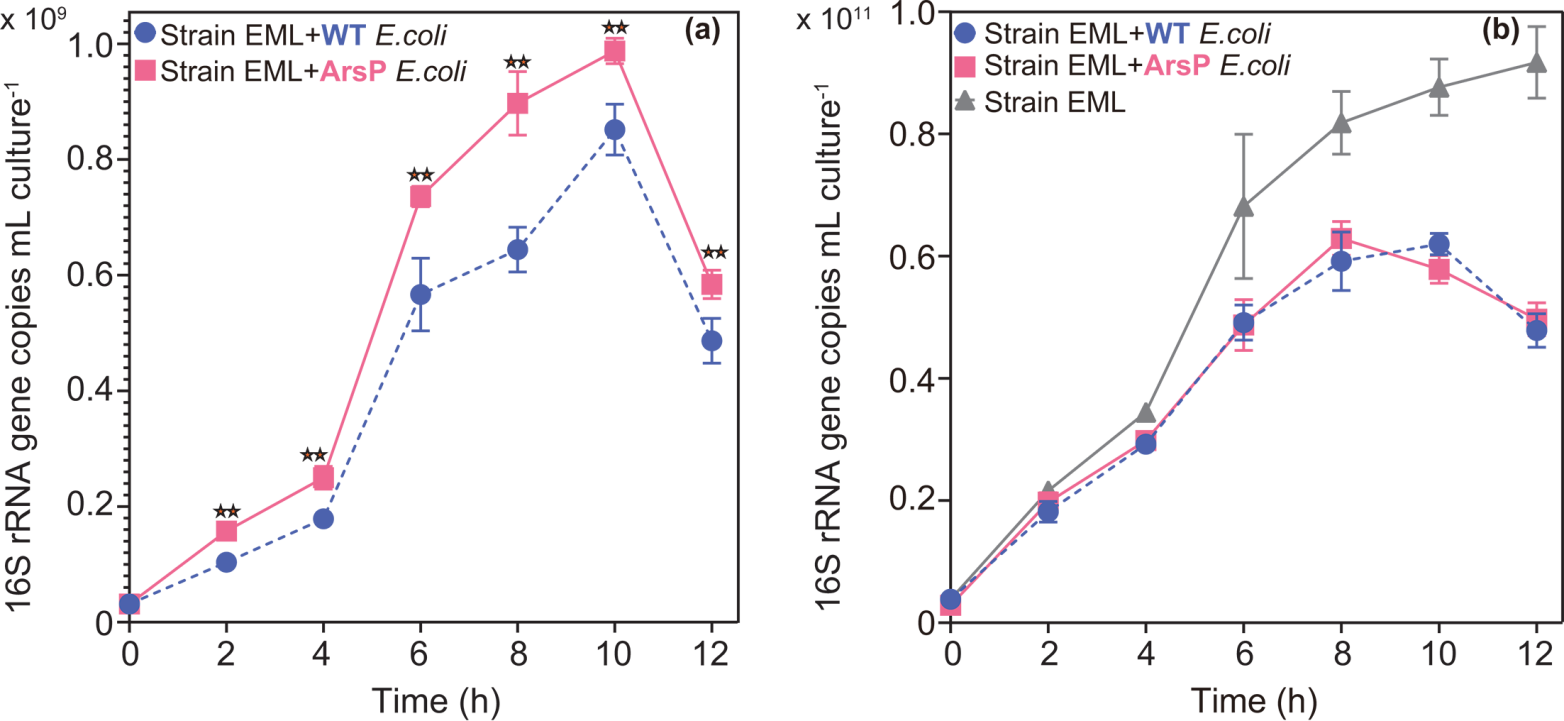
(**a**) Growth curves (16S rRNA gene copy number) of *Escherichia coli* K-12 wild-type strain MG1655 (WT *E. coli*) and engineered WT *E. coli* harboring a MMAs(III)-resistance gene (*arsP*) (ArsP *E. coli*) in anaerobic co-culture with *Paraclostridium bifermentans* strain EML in anoxic RCB with 25 µM iAs(III). (**b**) Growth curves (16S rRNA gene copy number) of *Paraclostridium bifermentans* strain EML in anaerobic co-culture systems as described above. Two-star symbols represent statistical significance at *P* < 0.01. Individual values for each biological replicate are shown in Data Table 4.

Next, we aimed to investigate the direct impact of As methylation by strain EML on other microorganisms. Confirmation of As(III)-resistance in *E. coli*, sensitivity to MMAs(III) in WT *E. coli*, and resistance to MMAs(III) in *E. coli* expressing *arsP* (hereafter, ArsP *E. coli*) is provided in Fig. S11 to S13 and Texts SR2-SR3. Additionally, the optimization of the co-culture ratio for incubations is detailed in Fig. S14 to S16 and Text SR4. WT or ArsP *E. coli* in anaerobic co-culture with strain EML exhibited similar growth patterns, showing rapid growth within 10 h of incubation, followed by a decline in copy number (Fig. 4a). Notably, the growth of ArsP *E. coli* was significantly higher (*P* < 0.01) than that of WT *E. coli* (Fig. 4a), while the growth of strain EML did not significantly differ between the two co-culture treatments (Fig. 4b). Thus, the growth difference between WT *E. coli* and ArsP *E. coli* in anaerobic co-culture with strain EML cannot be explained by growth rate differences in strain EML during the co-culture period. We propose that this difference in growth rates is due to the production of toxic MMAs(III), which inhibits the growth of WT *E. coli*, but has negligible effects on ArsP *E. coli* (Fig. S12 and S13). Furthermore, strain EML exhibited greater growth when cultured alone compared to its growth in co-culture with either strain of *E. coli* (Fig. 4b), which can be reasonably attributed to competition for growth substrates in co-culture.

### MMAs(III) production and *arsM* gene transcription during co-culture

**Fig 5:**
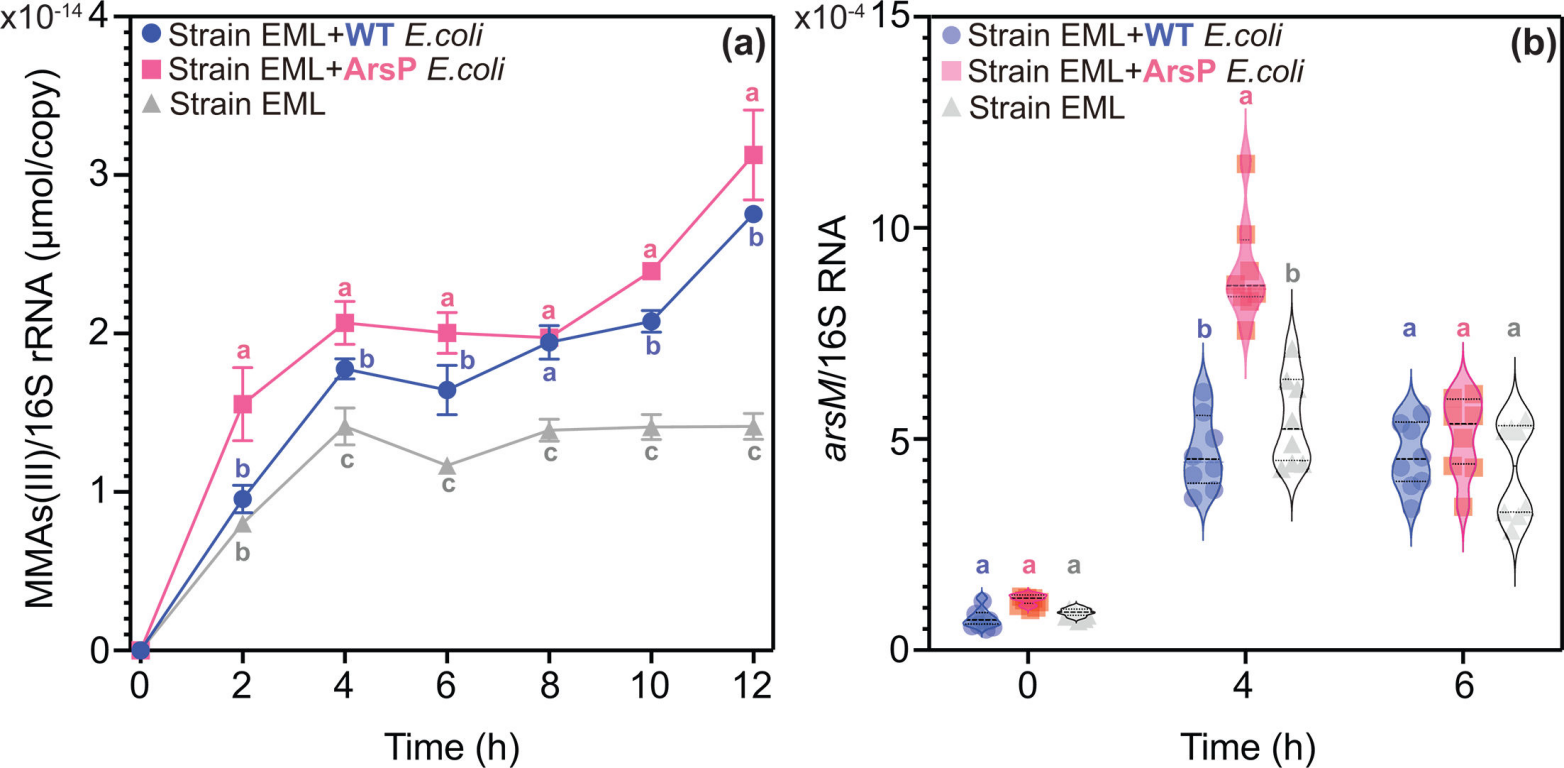
(**a**) Time-dependent concentration of aqueous MMAs(III) (normalized to 16S rRNA gene copies) in anaerobic co-culture *Paraclostridium bifermentans* strain EML with either WT *E. coli* or ArsP *E. coli* in anoxic RCB with 25 µM iAs(III). (**b**) Transcripts of *arsM* gene of strain EML (normalized to 16S rRNA gene copies) in anaerobic co-culture systems as described above at 0, 4, and 6 h of incubation. Different letters indicate significant differences at *P* < 0.05. Individual values for each biological replicate are shown in Data Tables 5 and 21.

From an ecological perspective, we would anticipate that strain EML would produce higher levels of MMAs(III) when co-cultured with ArsP *E. coli* compared to WT *E. coli*, as a strategy to thwart competition for nutrients with the faster-growing strain. Notably, MMAs(III) was identified as the dominant methylated As species, with its concentration gradually increasing alongside the decrease of iAs(III) throughout the co-culture period (Fig. S17). This is evidenced by the observation that MMAs(III) concentrations followed this trend upon normalization to biomass (16S rRNA gene copy number of strain EML): strain EML < strain EML + WT *E*. *coli* < strain EML + ArsP *E*. *coli* (Fig. 5a).

RT-qPCR was further employed to investigate how substrate competition influences the transcription of the *arsM* gene in anaerobic co-culture systems. The abundance of the transcribed *arsM* gene in strain EML was measured during the exponential growth phase (at 4 h and 6 h) in anoxic co-culture conditions (Fig. 5b). The transcription of the *arsM* gene in strain EML, normalized to its 16S rRNA gene copy number, was significantly (*P* < 0.05) higher when co-cultured with ArsP *E. coli* compared to WT *E. coli* at 4 h, corresponding to the mid-exponential phase (Fig. 5b). This difference in *arsM* gene expression aligns with the greater production of MMAs(III) observed in the co-culture of strain EML with ArsP *E. coli* compared to that with WT *E. coli* (Fig. 5a). We hypothesize that strain EML encounters more intense competition from ArsP *E. coli* than from WT *E. coli*, leading to a more rapid depletion of the growth substrates. This, in turn, would elevate *arsM* expression and ultimately enhance MMAs(III) production.

## DISCUSSION

In this study, we present evidence that trivalent monomethylated As (MMAs(III)) is produced as the predominant methylated As species by the recently isolated anaerobic As-methylating bacterium, *Paraclostridium bifermentans* strain EML, during its exponential growth phase in the presence of iAs(III) (Fig. 1 and 2). This finding strongly indicates that strain EML actively methylates As. This contrasts with several other anaerobic bacteria that possess a gene encoding a functional ArsM but do not exhibit active methylation (22). Additionally, an *arsM*-containing *ars* operon (*arsM-acr3-MPPE-arsR*1) identified in strain EML (Fig. S1) is presumed to include the ArsM encoding gene.

A major question remains: what is the ecological function of generating a product (MMAs(III)) that is more toxic than the substrate (iAs(III))? At first glance, this process may seem detrimental to the microorganism. However, it could lead to beneficial outcomes if two conditions are met. The first condition is that trivalent methylated As compounds can function as antibiotics, inhibiting the growth of competing microorganisms (21). Given that MMAs(III) and DMAs(III) are thermodynamically stable under anoxic conditions, they can persist long enough to serve effectively as antibiotics against anaerobic competitors. The second condition is that MMAs(III), produced intracellularly, must be exported to the extracellular environment, thereby preventing self-toxicity. If the rate of efflux of MMAs(III) exceeds that of iAs(III), then As methylation would represent a net detoxification process. The conditions that favor anaerobic As methylation remain elusive. A comparative study of As methylation among aerobic and anaerobic microorganisms have shown that, despite encoding a functional ArsM, anaerobes did not necessarily methylate iAs(III) (22). This observation has been partially attributed to the efficient efflux of iAs(III) in anaerobes (but not in aerobes), which prevents sufficient intracellular accumulation of iAs(III) for effective methylation. For instance, knocking out the *acr3* gene, which encodes the iAs(III)-specific efflux pump in the anaerobe *Clostridium pasteurianum*, resulted in a notable increase in intracellular iAs(III) but, yet there was only a marginal improvement in As methylation efficiency (22). A recent study indicated that knocking out the iAs(III) efflux pump gene *arsB* in *E. coli* cells expressing *arsM* led to increased As methylation under oxic conditions (30). While the influx of iAs(III) is a crucial factor controlling on As methylation, we propose that additional factors may influence anaerobic As methylation. If the microbial warfare hypothesis for anaerobic As methylation holds, it is possible that such methylation is triggered by environmental signals indicating obstacles to optimal growth (e.g., resource limitations) or by specific metabolites produced by other members of the microbial community, signaling their potential competiton (22, 24).

**Fig 6:**
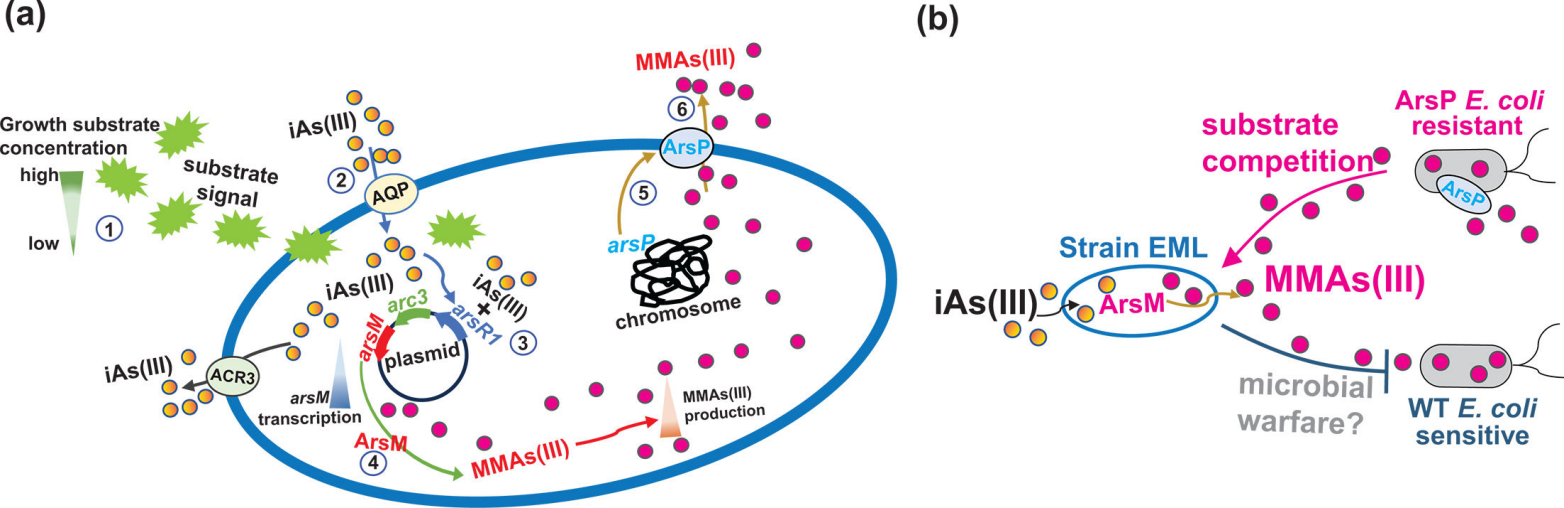
(**a**) Schematic of *Paraclostridium bifermentans* strain EML under anoxic growth substrate-limiting condition. Strain EML increases MMAs(III) production. Numbers denote steps in the response by strain EML: ① Receiving signal of substrate depletion, ② Entry of iAs(III) into cell, ③ Repressor ArsR1 binds to iAs(III) allowing transcription of *arsR*1, *acr*3, and *arsM* genes, ④ ArsM methylates iAs(III) to MMAs(III), ⑤ Transcription of *arsP* is activated (mechanism unknown) and ArsP produced, and ⑥ MMAs(III) is effluxed *via* ArsP. (**b**) Schematic of the interaction *P. bifermentans* strain EML with competing *E. coli*. Microbial inhibition and competition between MMAs(III)-producing strain EML and MMAs(III)-resistant and -sensitive *E. coli* strains under anoxic conditions.

Here, the first aim was to investigate the impact of substrate competition on anaerobic As methylation by generating growth substrate limitations. We observed opposing trends between growth substrate content and the concentration of aqueous MMAs(III) (Fig. 2) as well as *arsM* gene transcript numbers in the presence of iAs(III) (Fig. 3). Under conditions of limited growth substrates, strain EML produced higher (protein-normalized) concentrations of MMAs(III) (Fig. 2) and exhibited increased (protein-normalized) expression of the *arsM* gene (Fig. 3). This suggests that the cells responded to the limitation of growth substrates by enhancing As methylation. The underlying mechanism by which strain EML responds to growth substrate limitation through the regulation of As methylation remains unclear. A recent study on aerobic As methylation demonstrated that the uptake and methylation of iAs(III) are enhanced under low organic carbon conditions compared to high organic carbon conditions (31). The authors proposed that at elevated organic carbon levels, down-regulation of the *glpF* gene, which encodes an aquaglyceroporin channel facilitating the uptake of glycerol and iAs(III), results in decreased iAs(III) uptake. This finding provides insight into how strain EML may respond to substrate availability at the cellular level, as summarized in Fig. 6a. Briefly, substrate depletion likely activates the As methylation system due to the increased intracellular availability of iAs(III). This response specifically involves up-regulation of the *arsM* expression, leading to increased production of MMAs(III). Thus, increased methylation appears to be an indirect response to substrate limitation *via* elevated intracellular iAs(III). Consistent with this interpretation, we noted the accumulation of intracellular iAs(III) preferentially under low-growth substrate conditions, confirming the close relationship between intracellular iAs(III) levels and high As methylation potential, as previously reported (22). In contrast to the aerobic strains discussed in the aforementioned study, strain EML possesses the *acr3* and *arsP* genes. We propose that the repression or inhibition of the iAs(III) efflux permease, encoded by the *acr3* gene, may occur in strain EML under substrate-limited conditions. This implies that a less efficient iAs(III) efflux system leads to increased intracellular iAs(III) buildup in the cytoplasm, thereby facilitating enhanced anaerobic As methylation. Furthermore, the expression of the MMAs(III) efflux permease gene, *arsP,* could also be up-regulated, enabling efficient extrusion of MMAs(III) into the environment to prevent self-toxicity and promote As methylation.

Next, direct evidence of microbial inhibition through the production of MMAs(III) was sought by the anaerobic co-culture of strain EML with either MMAs(III)-sensitive WT *E. coli* or MMAs(III)-resistant *E. coli* (designated as ArsP *E. coli*) in RCB medium (Fig. 4 and 5). Our finding revealed a significant two-way interaction between strain EML and *E. coli*, as outlined in Fig. 6b. Specifically, the MMAs(III) produced by strain EML inhibited the growth of WT *E. coli* more effectively than that of ArsP *E.coli* (Fig. 4 and 6b), thereby supporting the microbial warfare hypothesis. Conversely, we interpret the reduced growth of WT *E. coli* as resulting in less substrate depletion compared to ArsP *E. coli*, leading to lower expression of the *arsM* in strain EML (Fig. 5 and 6b). However, it is also plausible that other factors, such as signaling mechanisms, could contribute to the observed reduction in *arsM* expression. Therefore, we can reasonably conclude that the production of MMAs(III) serves to inhibit microorganisms that are not equipped to detoxify it, while strain EML increases *arsM* expression and, consequently, MMAs(III) production in response to substrate limitation. Notably, although more MMAs(III) is produced by strain EML when co-cultured with ArsP *E. coli* than WT *E. coli*, the growth of strain EML remains comparable in the presence of either *E. coli* strain (Fig. 4b). This observation can be explained by the idea that while the production of MMAs(III) is a response to substrate limitation, it does not sufficiently deter the competitor (i.e., the prey) to impact resource utilization and overall growth. Indeed, the effects of resource competition are evident when comparing the growth of strain EML in the presence or absence of *E. coli* (Fig. 4b).

The efflux of MMAs(III) is essential for the effective delivery of this antibiotic to other microorganisms and is critical for avoiding self-toxicity, as outlined in condition 2 above. Specific and nonspecific efflux permease genes for MMAs(III), such as *arsP* (32) and *arsK* (33), are well-documented. The co-evolution of *arsM* and *arsP* has been previously evidenced, indicating a strategic mechanism for the efflux of MMAs(III) by both MMAs(III)-producing and MMAs(III)-resistant microorganisms (18–20). The identification of two chromosomally encoded *arsP* genes in strain EML suggests that it may possess the capability to effectively export MMAs(III) into the extracellular environment (Fig. S18). The variable expression of *arsP* across different conditions (althouh not measured in our study) may help explain the observed similarity in the levels of *arsM* expression when strain EML is grown with WT *E. coli* compared to when it is grown alone.

In this study, we provide direct evidence for the role of growth substrate competition in anaerobic As methylation by strain EML. Furthermore, we evidence a feedback loop in which a bacterium resistant to MMAs(III) enhances its production, presumably through enhanced expression of the *arsM* gene as a result of substrate limitation. This work uncovers the complex interactions between an anaerobic As methylator and its potential competitors. However, it is important to note that, in addition to substrate limitation, other unidentified signals and factors within the rice-soil system may also contribute to the elevated anaerobic As methylation observed in flooded paddy fields. Further research is necessary to confirm that the regulation of *arsM* gene expression by the growth substrate concentration is mediated by intracellular iAs(III) levels, as well as to identify additional factors that may influence anaerobic As methylation. A substantial understanding of the mechanisms controlling anaerobic As methylation is essential for developing effective strategies to limit As methylation in rice paddy soils.

## MATERIALS AND METHODS

### Growth experiment

The anaerobic As-methylating bacterium, *Paraclostridium bifermentans* strain EML (hereafter referred to as strain EML) was previously isolated from an anaerobic paddy soil enrichment (24, 34). To investigate how the availability of growth substrates affects As methylation activity, dilutions (vol/vol) of Reinforced Clostridial Broth (RCB) (Oxoid Ltd) in Milli-Q water were prepared in 120 mL serum bottles containing 50 mL of medium (100% RCB, 75% RCB, 50% RCB, and 25% RCB) (Table S1). The medium was boiled for 5 min to remove O_2_, then cooled under a flow of 100% N_2_ gas to room temperature and dispensed into individual culture serum bottles under the same N_2_ atmosphere. The bottles were sealed with sterile rubber stoppers and crimped with aluminum caps, and the headspace was flushed with 100% N_2_ to ensure anaerobic conditions before autoclaving at 121°C for 15 min.

A pre-culture of strain EML was grown anaerobically in RCB to mid-exponential growth phase. Approximately 0.5 mL of strain EML was inoculated into each RCB dilution containing 25 µM iAs(III) as sodium arsenite (or into the equivalent no-iAs(III) control) in triplicate using sterile N_2_-flushed syringes and needles. The inoculum represented approximately 1% of the total volume (vol/vol). All bottles were incubated at 30°C in the dark without shaking. A total of eight experimental conditions were selected (Table S3). At selected time points and for each condition, triplicate bottles were sampled for growth quantification, which was assessed using both optical density at 600 nm (OD_600_) and total protein content estimated using a BCA protein assay kit (Thermo Scientific, MA, USA). For quantification of *arsM* gene expression, triplicate cultures were sampled for RNA extraction at 8 and 24 h. To assess the stability of MMAs(III) in RCB medium, additional abiotic control experiments (including fresh/spent RCB medium supplemented with MMAs(III) and chemical reaction between MMAs(III) and sulfide) were performed in duplicate (see Text Methods SM1 in the supplemental material).

### Arsenic speciation

At each time point and for each condition, aqueous and intracellular As speciation and total As were measured. Samples for aqueous As species and total As were obtained from 1 mL of culture collected using sterile, N_2_-flushed syringes and needles. These samples were then filtered through 0.22 µm cellulosic membrane filters and stored in 1 mL of 1% HNO_3_ (≥69%, Honeywell Fluka). In addition, to analyze As species and total As post-sample oxidation, another 1 mL of culture was obtained as described above and oxidized by adding 10% (vol/vol) hydrogen peroxide (wt/vol) (H_2_O_2_, 30%, Reactolab SA) and stored overnight in a 1% HNO_3_ solution. For soluble intracellular As species, 1 mL of culture was collected, and the cells were pelleted by centrifugation at 8,000 × *g* for 5 min, then stored at −20°C until further use. To release soluble intracellular As, the cell pellets were lysed in a lysis buffer (0.1% Triton X-100, 0.1% SDS, 10 mM EDTA, and 1 mM Tris-HCl) at 95°C for 15 min, with vortexing every 3 min (22). The lysed cell suspension was subsequently centrifuged at 8,000 *g* for 5 min, and the pellet was resuspended in 200 µL 1× PBS buffer for protein determination, as described above. The supernatant was filtered through 0.22 µm filters and reserved for As speciation and total As analysis.

Both aqueous and soluble intracellular As speciation were determined using high-performance liquid chromatography and inductively coupled plasma mass spectrometry (HPLC-ICP-MS) on an Agilent 8900 ICP-QQQ instrument. A previously described anion exchange protocol utilizing step-gradient elution mode with an As Spec anion exchange fast column (50 mm × 4.0 mm, PrinCen, Guangzhou, China) was employed.^21^ Six As standards were prepared: MMAs(III) as methyldiiodoarsine (Santa Cruz Biotechnology Inc.), TMAs(V)O as trimethyl arsine oxide (Argus Chemicals Srl., Italy), DMAs(V) as sodium dimethylarsinate (ABCR, Germany), MMAs(V) as monomethylarsonic acid (Chemservice, PA, USA), As(III) as sodium arsenite (NaAsO_2_) (Sigma-Aldrich, MO, USA), and As(V) as sodium arsenate dibasic heptahydrate (Na_2_HAsO_4_·7H_2_O) (Sigma-Aldrich, MO, USA). Additionally, monomethylmonothioarsonic acid (MMMTAs(V)) was synthesized as previously described (Text SM1) (35). Total aqueous and soluble intracellular As concentrations were quantified using the same ICP-MS instrument operated in stand-alone mode (24).

### RNA extraction and RT-qPCR

Each culture sample was treated with RNAprotect bacterial reagent (Qiagen, Hilden, Germany) following the manufacturer’s recommendations to stabilize RNA and prevent degradation. RNA was extracted using the RNeasy Mini Kit (Qiagen), following the manufacturer’s instructions and an initial sample preparation protocol outlined in the Qiagen RNAprotect bacteria reagent handbook. Protocol 5 (enzymatic lysis, proteinase K digestion, and mechanical disruption of bacteria) was employed for cell lysis. Genomic DNA digestion was completed during RNA purification using the RNase-Free DNase set (Qiagen). Reverse transcription was performed with QuantiTect reverse transcription kit (Qiagen). Detailed information on the design of the *arsM* gene primer set, optimization of PCR amplification conditions, and construction of an *arsM* plasmid for generating a standard curve are presented in Text SM2. The RT-qPCR was conducted using a Mic PCR system (Bio Molecular Systems, Mic) using SYBR Green Master Mix. The reactions were performed in a total volume of 10 µL, containing 5 µL of the 2× SensiFAST SYBR No-ROX Kit (Bioline, London, UK), 0.2 µM of each *arsM* gene primer, 2.5 µL of cDNA, and 1% (vol/vol) bovine serum albumin (BSA) (Sigma). A 10-fold dilution series containing 10^7^–10^1^ copies of strain EML *arsM* plasmid DNA was used to generate a standard curve. All samples were run in quadruplicates. An NRT (no-reverse transcriptase control) and an NTC (no template control) were both included as negative controls.

### Quantification of *arsM* gene transcripts

To get a comprehensive understanding of *arsM* gene expression under variable substrate conditions, both relative and absolute quantification methods were attempted. For relative quantification, the specific primer sets for the housekeeping genes utilized and their corresponding amplification conditions are presented in Table S2. However, these genes are not employed in this study as they did not satisfy the minimum criteria for a stable reference gene (refer to the results in Section on *arsM* gene transcription). For absolute quantification, we were concerned that the mRNA yield would be variable across conditions due both to biological reasons (e.g., rate and extent of growth) and biases introduced by RNA extraction. To obtain reliable results, we compared two methods of absolute quantification. The first method involved adjusting biomass for each culture prior to RNA extraction to ensure that RNA was extracted from the same amount of biomass (OD_600_) regardless of conditions. The same volume of total RNA was used for reverse transcription, and the expression data (*arsM* copy numbers) were presented relative to OD_600_ (biomass). The second method involved quantifying the extracted total RNA and using the same amount of RNA from all conditions for reverse transcription. The expression data were subsequently normalized to the corresponding protein concentration.

### Anaerobic co-culture system

To provide direct evidence of the effects of substrate competition among microbes and the associated microbe-microbe interactions on anaerobic As methylation, anaerobic co-culture systems (predator-prey systems) were established. The “predator” was strain EML (24). One “prey” was wild-type *Escherichia coli* K-12 strain MG1655 (WT), which is sensitive to MMAs(III) (Fig. S12 and S13) and resistant to As(III) (36) (Fig. S3; Text SM3). The other was the same strain in which the *arsP* gene was integrated into the chromosome by the mini-Tn7-based gene integration method (37) and controlled by the promoter from the *fnr*S gene (Texts SM4-SM6), a highly conserved small RNA that is induced under anaerobic conditions (38). The *arsP* gene encodes ArsP, a MMAs(III) efflux permease responsible for extruding trivalent organoarsenicals from the cells (32). Expression of *arsP* in *E. coli* confers resistance to MMAs(III) under anoxic conditions. The confirmation of As(III) resistance in WT *E. coli* and in *E. coli* expressing *arsP* (hereafter referred as ArsP *E. coli*), along with MMAs(III) sensitivity in WT *E. coli*, and MMAs(III) resistance in ArsP *E. coli,* is illustrated in Fig. S11 to S13 and detailed in Text Results SR2-SR3 in the supplemental material.

Co-culture treatments were conducted in triplicate as described above in anaerobic serum bottles containing 50 mL of 100% RCB medium, consisting of (i) strain EML + WT *E*. *coli* + 25 µM iAs(III), (ii) strain EML + ArsP *E*. *coli* + 25 µM iAs(III), or (iii) a control with only strain EML + 25 µM iAs(III). Due to the difference in growth rates between strain EML and *E. coli*, various inoculation ratios between the co-culture members were tested, with the optimal ratio determined to be 10% EML (vol/vol), that is, the cell pellet from a 5 mL of exponential phase culture of strain EML in RCB and 50 µL of exponential phase culture of *E. coli* (WT or ArsP *E. coli*) in 50 mL of RCB (Fig. S14 to S16; Texts SM7 and SR4). This co-culture cell ratio was selected to ensure that a sufficient number of strain EML cells were present to produce at least 1 µM MMAs(III); otherwise, *E. coli* would dominate the co-culture due to their distinct growth rates. During the anaerobic co-culture period, aqueous As speciation, growth rate, and *arsM* gene transcripts were measured using HPLC-ICP-MS, qPCR (quantifying the 16S rRNA gene copy numbers of strain EML and the two *E. coli* strains), and RT-qPCR, respectively.

## SUPPLEMENTAL MATERIAL

Supplemental material aem.00961-24-s0001.docx Supplemental methods and results, Fig. S1 to S18, and Tables S1 to S3.

Raw data aem.00961-24-s0002.xlsx All available raw data associated with the paper.

ASM does not own the copyrights to Supplemental Material that may be linked to, or accessed through, an article. The authors have granted ASM a non-exclusive, world-wide license to publish the Supplemental Material files. Please contact the corresponding author directly for reuse.
